# The Extent of Synaptic Stripping of Motoneurons after Axotomy Is Not Correlated to Activation of Surrounding Glia or Downregulation of Postsynaptic Adhesion Molecules

**DOI:** 10.1371/journal.pone.0059647

**Published:** 2013-03-19

**Authors:** Alexander Berg, Johan Zelano, Sebastian Thams, Staffan Cullheim

**Affiliations:** 1 Department of Neuroscience, Karolinska Institutet, Stockholm, Sweden; 2 Department of Neuroscience, Uppsala University, Uppsala, Sweden; University of Sydney, Australia

## Abstract

Synapse elimination in the adult central nervous system can be modelled by axotomy of spinal motoneurons which triggers removal of synapses from the cell surface of lesioned motoneurons by processes that remain elusive. Proposed candidate mechanisms are removal of synapses by reactive microglia and astrocytes, based on the remarkable activation of these cell types in the vicinity of motoneurons following axon lesion, and/or decreased expression of synaptic adhesion molecules in lesioned motoneurons. In the present study, we investigated glia activation and adhesion molecule expression in motoneurons in two mouse strains with deviant patterns of synapse elimination following axotomy. Mice deficient in complement protein C3 display a markedly reduced loss of synapses from axotomized motoneurons, whereas mice with impaired function of major histocompatibility complex (MHC) class Ia display an augmented degree of stripping after axotomy. Activation of microglia and astrocytes was assessed by semiquantative immunohistochemistry for Iba 1 (microglia) and GFAP (astrocytes), while expression of synaptic adhesion molecules was determined by in situ hybridization. In spite of the fact that the two mouse strains display very different degrees of synapse elimination, no differences in terms of glial activation or in the downregulation of the studied adhesion molecules (SynCAM1, neuroligin-2,-3 and netrin G-2 ligand) could be detected. We conclude that neither glia activation nor downregulation of synaptic adhesion molecules are correlated to the different extent of the synaptic stripping in the two studied strains. Instead the magnitude of the stripping event is most likely a consequence of a precise molecular signaling, which at least in part is mediated by immune molecules.

## Introduction

Peripheral axotomy triggers the removal of synapses from lesioned motoneurons by mechanisms that still remain elusive [1]–[5]. The ‘synaptic stripping’ after axotomy is accompanied by a vivid glial response in the spinal cord. Thus, within a few days after axotomy, astrocytes and microglia are activated in the vicinity of the lesioned motoneurons and glial processes are inserted between synaptic boutons and the motoneuron surface in a manner suggesting an active synaptic removal by glial cells [1]–[4], [6]. Based on ultrastructural observations, microglia has been suggested to have a key role in the elimination process [2]. Subsequent studies have shown that pharmacological or genetical ablation of the injury-induced proliferation of microglia does not affect synaptic stripping of axotomized motoneurons [1], [7], [8], leading to the suggestion that astrocytes might be involved in the synaptic stripping process.

Another possible mechanism for the reduced number of synaptic inputs to axotomized motoneurons is an attenuated adhesiveness between the motoneurons and presynaptic terminals [1]. Synaptic adhesion molecules do not only preserve the structural integrity of synapses, but some of them can also regulate the formation and elimination of synapses. Expression of SynCAM1, neuroligin (NLG)-2, -3 and netrin G-2 ligand (NGL-2) induces synapse formation, while various forms of interference with their normal neuronal expression inhibit synapse formation [9]–[14]. These molecules are all downregulated in axotomized motoneurons before the loss of synapses and their expression return as new synapses are formed [6], [15], [16].

Recently, certain groups of molecules with effects on the stripping response have been identified, such as the major histocompatibility complex (MHC) class I molecules and members of the complement family. Mice with impaired function of several MHC molecules exhibit a greater degree of synaptic stripping after axotomy [17], while complement protein C3 deficient mice show a much less pronounced degree of synaptic stripping compared to WT mice [18]. This suggests that immune molecules may regulate both the shedding and retention of synapses. These two genetically modified mouse strains thus constitute intriguing model systems to study whether MHC class I and C3 regulated synapse plasticity are dependent on either one of the two basic mechanisms proposed for synaptic removal [1].

Thus, we hypothesized that the differences in synaptic stripping between the strains could be due to a modulated glial response, with a differential activation of microglia and/or astrocytes. Alternatively, the effects could be exerted by an influence on the downregulation of synaptic adhesion molecules in the lesioned motoneurons.

We have here investigated the glial activity response by use of the markers, glial fibrillary acidic protein (GFAP) in astrocytes and Iba1 in microglia, as well as the regulation of the synaptic adhesion molecules SynCAM1, NLG-2, -3 and NGL-2 after sciatic nerve lesion in mice with an altered synaptic stripping following motoneuron axotomy. Based on our previous studies, we used the following two animal models for these studies - K^b−/−^D^b−/−^ mice, lacking classical MHC class I (MHC class Ia) and C3^−/−^ mice, lacking the complement protein C3.

## Materials and Methods

### Animals Experiments

K^b−/−^D^b−/−^ and C3^−/−^ mice have previously been described [19]–[23]. Briefly, K^b−/−^D^b−/−^ and C3^−/−^mice were bred from different mouse lines of C57BL6 mice. K^b−/−^D^b−/−^ mice were a kind gift from F. A. Lemonnier (Pasteur Institute, Paris, France) and were bred and maintained at the Department of Microbiology, Tumor and Cell Biology (Karolinska Institutet, Stockholm, Sweden). C3^−/−^ mice were a kind gift from associate professor Marcela Pekna (Sahlgrenska University, Gothenburg). Mice with a silencing mutation of the C3 gene were generated by the gene targeting in embryonic stem (ES) cells approach and was then transported to Karolinska Institutet for experiments. Young adult female K^b−/−^D^b−/−^ (n = 11) and C3^−/−^ (n = 11) and age matched WT (n = 17) females were anesthetized with isoflurane. K^b−/−^D^b−/−^ and WT from the same litters were operated in one experiment and C3^−/−^mice and WT controls from the same litters in another experiment. Postoperatively mice were given a s.c. injection of Temgesic. The left sciatic nerve was transected at the obturator tendon level. A 2–4 mm long segment of the distal portion of the nerve was removed in order to prevent regeneration. After a survival time of 7 days mice were subjected to lethal carbon dioxide inhalation.

### In situ Hybridization

After decapitation, the lumbar section of the spinal cord was rapidly dissected. The lumbosacral intumescence was promptly frozen and sectioned at −20°C in a cryostat (Microm HM 500M, Heidelberg, Germany) in 12 µm-thick transverse sections, which were stored at −20°C until used.

Oligonucleotides were synthesized (CyberGene AB, Huddinge, Sweden) and *in situ* hybridization (ISH) was performed as previously described [24]. The sequences of the probes were checked in a GeneBank database search to exclude significant homology with other genes. The probes used were NLG-2 (NM_053992, nucleotides 2606–2653), NLG-3 (NM_134336, 919–966), SynCAM1 (BC078966, 716–763), NGL-2 ((DQ_119102.1, 2933–2980 and 2922–2969). To ensure specificity we synthesized control probes directed against a different portion of the target mRNA and verified that these probes rendered signal patterns identical to those from the probes used for semiquantification. Furthermore, control tissues with known expression of the studied molecules were used to ensure specificity of the probes.

The P^33^ labelled probes were labelled at the 3′-end with deoxyadenosine-alpha-triphosphate and hybridized to the sections without pre-treatment for 16–18 hours at 42°C. The hybridization mixture contained 50% formamide, 4×SSC (1×Standard Saline Citrate = 0.15 M NaCl and 0.015 M sodium citrate), 1×Denhardt’s solution, 1% sarcosyl (N-lauroylsarcosine; Sigma), 0.02 M phosphate buffer, 10% dextran sulphate (Pharmacia), 250 µg/ml yeast tRNA (Sigma), 500 µg/ml sheared and heat-denaturated salmon sperm DNA (Sigma), and 200 mM dithiothreitol (DTT; LKB, Bromma, Sweden). Following hybridization, the sections were washed several times in 1×SSC at 55°C, dehydrated in ethanol, and dipped in NTB2 nuclear track emulsion (Kodak, Rochester, NY). After 3 weeks, the slides were developed in D-19 developer (Kodak), counterstained (Xylen) and coverslipped. The sections were examined in a Leica DM RBE microscope (Leica, Wetzlar, Germany), equipped with a dark field condenser and appropriate filter to examine ultraviolet fluorescence. Images were captured with a Nikon CoolPix 990 camera (Nikon, Tokyo, Japan).

Semiquantitative measurements of the ISH signal were carried out as previously described [25]. Briefly, the mRNA hybridization signal overlaying motoneuron cell bodies, identified by their size and location in the sciatic motor column, or the signal over the entire motoneuron pool was recorded with a PL-APO objective in the Leica microscope and digitized using a Kappa video camera (Mikroskop System; Näsviken, Sweden). The gray scale of the dark field image was adjusted and segmented using the enhance contrast and density slicing feature of the NIH Image software (version 1.55; NIH; Behesda, MD, USA) so that the silver grains were assessed automatically. The signal over the ipsilateral (IL) motoneuron pool was compared to the corresponding contralateral (CL) area in the same spinal cord section. At least four spinal cord sections from each animal were measured and the mean IL/CL ratio for each animal was used for statistical analysis (n = 5–8 per group).

### Immunohistochemistry

Animals used for immunohistochemistry were transcardially perfused with Tyrode’s solution 30 seconds followed by Lana’s fixative (4% Formalin and 0.4% picric acid in 0.16 M PBS, pH 7.2) at 20°C. The spinal cords were rapidly dissected and kept in the same fixative for overnight at 4°C, rinsed, and stored 24 h in 10% sucrose with 0.1% sodium azide in 0.01 M PBS at 4°C for cryoprotection. The tissues were sectioned with a cryostat into 14 µm thick sections.

Sections were incubated overnight at 4°C with primary antisera (synaptophysin 1∶200, Zymed raised in rabbit, Iba1 1:1000, Wako, raised in rabbit and GFAP 1∶200, Sigma-Aldrich, raised in rabbit) in 0.01 M PBS with 5% donkey serum and 0.3% Triton X-100, thereafter rinsed in PBS 0.01 M and incubated for 60 min with alexa-488 conjugated secondary antibody, diluted in PBS and 0,3% Triton X-100 rinsed in PBS and mounted in PBS-glycerol (1∶3). All antibodies were suitable for IHC on mouse tissue according to the manufacturers and previously described [26]–[28]. As negative control, non-immunized rabbit serum was used instead of primary antibody, which abolished the staining.

After immunohistochemistry, sections were examined in a Zeiss LSM 5 Pascal confocal laser scanning microscope (Carl Zeiss GmbH, Göttingen, Germany), equipped with argon/HeNe lasers. Cy-2 was visualized with 488 nm.

Semiquantative measurements for immunoreactivity were carried out in ImageJ (NIH) on confocal images of the sciatic motoneuron pool or nerve. The immunoreactivity in an area containing the injured sciatic motoneuron pool was compared to an area of the same size containing the contralateral uninjured sciatic motoneuron pool in the same spinal cord section. Images were taken in the optical plane with maximal immunoreactivity and all settings for compared sections were identical. At least four spinal cord sections were measured. For spinal cords the mean IL/CL ratio was used for statistical analysis. IHC on WT and K^b−/−^D^b−/−^, and WT and C3^−/−^mice was performed separately. C3^−/−^and K^b−/−^D^b−/−^ IR for GFAP and Iba1 were normalized to respective WT control and then compared.

Statistical analysis was performed in GraphPad Prism software (version 5.0; GraphPad software Inc., San Diego, CA, USA). P<0.05 was considered as significant. For quantification of immunohistochemistry and in situ hybridization four spinal cord sections from each animal were counted and the mean IL/CL ratio for each animal was used for statistical analysis. For comparison between two groups student’s t-test was performed and for multiple comparisons, one-way ANOVA with Bonferroni’s multiple comparison test was used. All analysis was conducted blinded for the observer.

## Results

**Figure 1:**
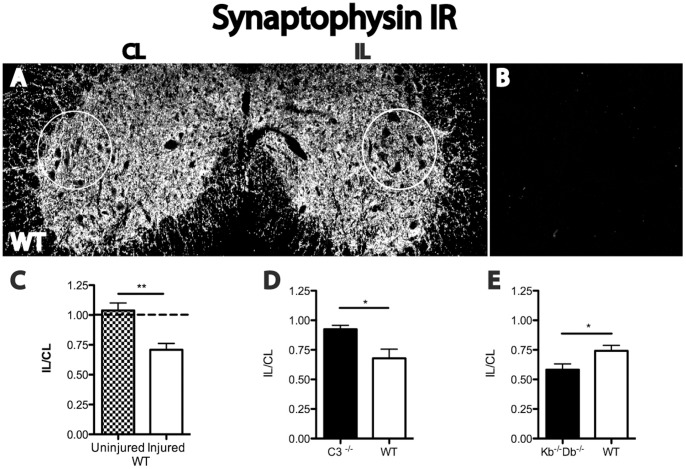
Reduced synaptic stripping in *C3^−/−^* mice and augmented stripping in K^b−/−^D^b−/−^ mice compared to WT control one week after sciatic nerve lesion. Immunoreactivity (IR) for synaptophysin in the sciatic motoneuron pool of WT mice (A). Control staining with primary antibody omitted and replaced by non-immunized rabbit serum (B). Ipsilateral (IL) to contralateral (CL) ratio of semiquantative measurements of synaptophysin IR was quantified for WT (C), *C3^−/−^* (D) and K^b−/−^D^b−/−^ (E). For WT unlesioned mice the IL/CL ratio was 1.04±0.07 compared to 0.71±0.05 for the lesioned mice (C). For *C3^−/−^* mice synaptophysin IR IL/CL ratio was decreased to 0.92 and for WT to 0.68 (D) and for K^b−/−^D^b−/−^ IL/CL ratio to 0.55 and for WT 0.74 (E). Six animals were used in each group. Error bars indicate SEM, * = p<0.05, unpaired t-test. Scale bar = 50 µm.

First, we compared the degree of synaptic detachment from motoneurons one week after sciatic nerve lesion between wild type (WT), K^b−/−^D^b−/−^ and C3^−/−^ animals. The two genetically modified mouse strains were compared to their individual WT controls. Semiquantitative measurements of immunoreactivity (IR) for the general presynaptic marker synaptophysin were performed, and the signal for the ipsilateral (IL) ventral horn was expressed as percent of the contralateral (CL) side (fig. 1A). No background staining was seen when primary antibody was omitted and replaced by non-immunized rabbit serum (fig. 1B).

WT mice displayed a reduction in synaptophysin IR IL/CL ratio from 1.04±0.07 to 0.71±0.05% one week after a sciatic nerve transection (SNT). (fig 1A–B). In lesioned C3^−/−^ mice, synaptophysin IR IL/CL ratio was estimated to 0.93±0.03 compared to 0.68±0.08 in WT mice, thus showing a significantly attenuated synaptic stripping in absence of C3 molecules (fig. 1C). In contrast, K^b−/−^D^b−/−^ mice displayed an enhanced axotomy-induced reduction in synaptophysin IR IL/CL ratio (0.58±0.05), compared to WT (0.74±0.05) mice (fig. 1D). Ultrastructural data supporting the large differences in synaptic stripping of cell bodies of axotomized motoneurons as indicated here by the synaptophysin data have been published previously [17], [18].

**Figure 2:**
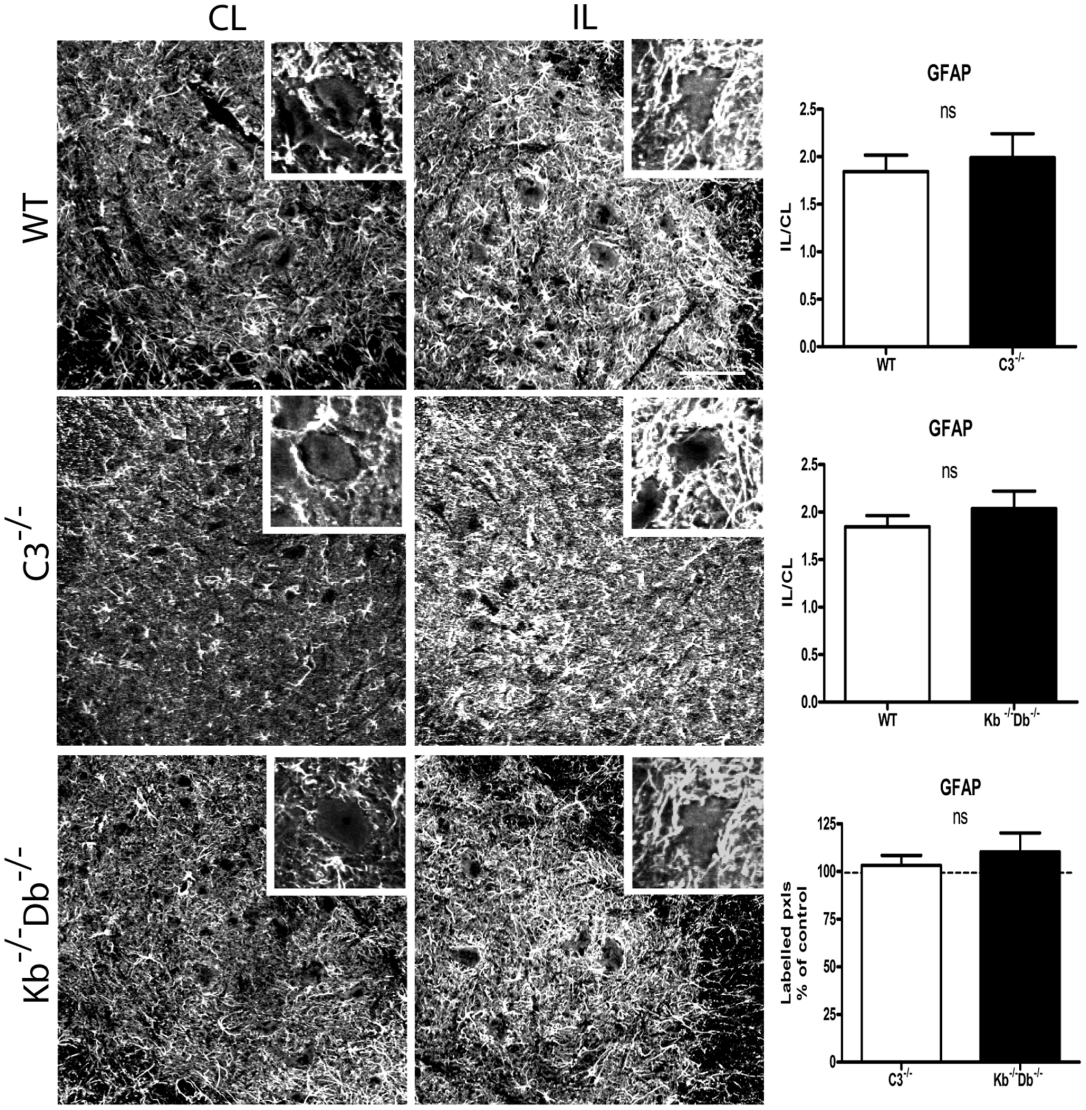
No difference in astrocyte activation as measured by GFAP IR between WT, C3^−/−^ and K^b−/−^D^b−/−^ mice one week after sciatic nerve lesion. Immunoreactivity for GFAP was measured in the sciatic motoneuron pool for WT mice (first row), C3^−/−^ (second row) and K^b−/−^D^b−/−^ mice (third row). Ipsilateral (IL, second column) to contralateral (CL, first column) ratio of semiquantative measurements was quantified in the third column. In WT mice, GFAP IR IL/CL ratio was increased to 1.99 and 1.85 of the value on the control side in the two studied sets of experiment. In C3^−/−^ mice the corresponding increase in IL/CL ratio was 1.84, and in K^b−/−^D^b−/−^ mice 2.04. Insets showing 50× magnification micrographs of GFAP IR around individual motoneurons. Six animals were studied in each group. Error bars indicate SEM, unpaired t-test. Scale bar = 50 µm.

We next studied changes in GFAP IR in the spinal cord, in order to assess astrocytic activation after lesion in the different mouse strains. One week after SNT we observed an upregulation of GFAP on the ipsilateral side compared to the contralateral side, particularly in the area surrounding lesioned motoneurons. The IL/CL ratio for GFAP-IR in C3^−/−^ mice was 1.99±0.25 compared to its WT control 1.84±0.17 (fig. 2A). For K^b−/−^D^b−/−^ mice the ratio was 2.04±0.18 compared to its WT control 1.85±0.12 (fig. 2B). No significant differences were seen between the strains (fig. 2C). Thus, we conclude that the absence of either one of the studied immune molecules did not grossly affect the degree of astrocyte reactivity in the spinal cord after a peripheral nerve lesion.

**Figure 3:**
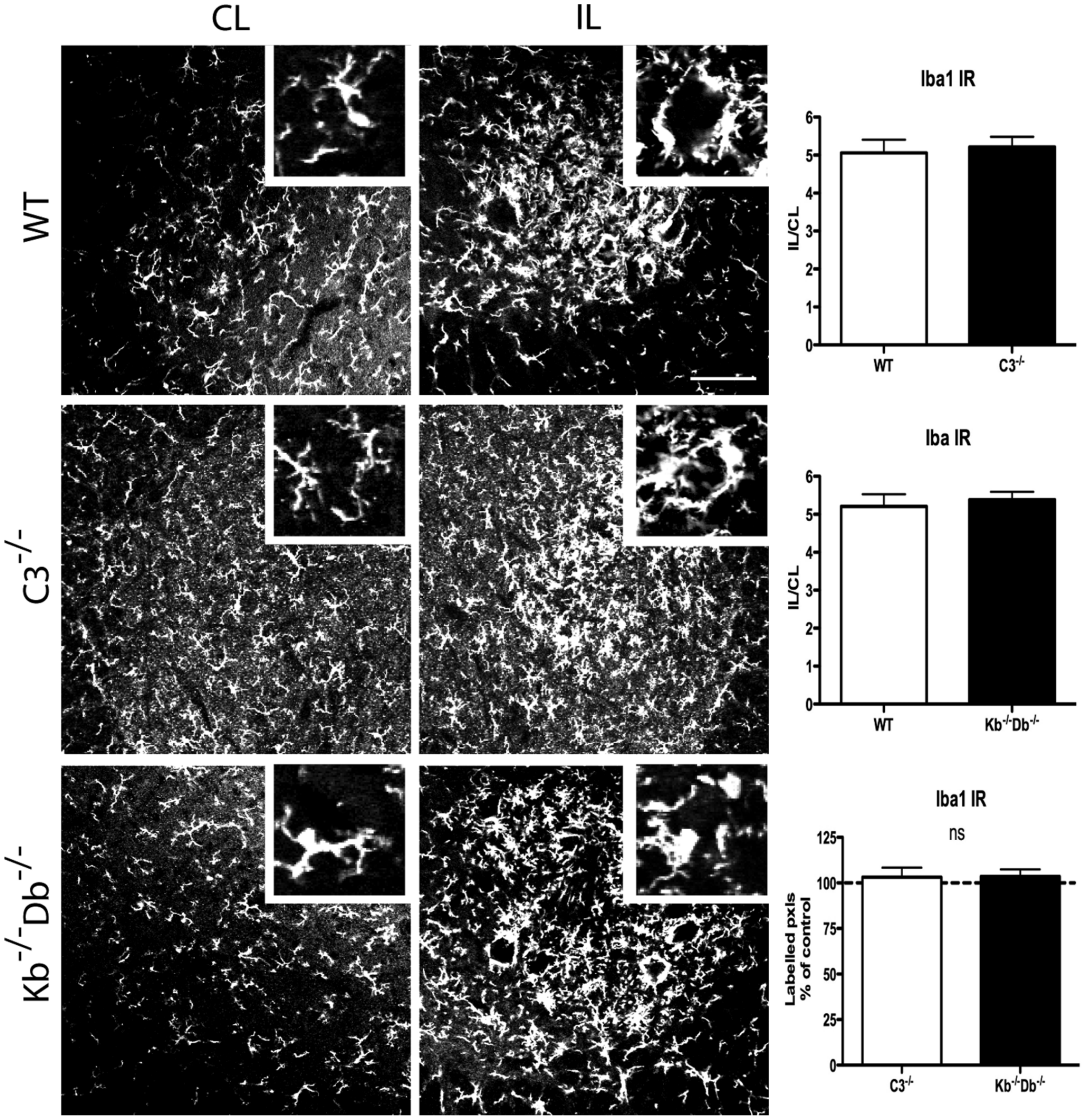
No difference in microglia activation as measured by Iba1 IR between WT, C3^−/−^ and K^b−/−^D^b−/−^ mice one week after sciatic nerve lesion. Immunoreactivity for Iba1 was measured in the sciatic motoneuron pool in WT (first row), C3^−/−^ (second row) and K^b−/−^D^b−/−^ mice (third row). Ipsilateral (IL, second column) to contralateral (CL, first column) ratio of semiquantative measurements was quantified in the third column. In WT mice, Iba1 IR IL/CL ratio was increased to 5.22 and 5.21 of the value on the control side in the two studied sets of experiment. In C3^−/−^ mice the corresponding increase in IL/CL ratio was 5.05, and in K^b−/−^D^b−/−^5.39. Insets indicate 50× magnification micrographs of Iba1 IR around individual motoneurons. Six animals were studied in each group. Error bars indicate SEM, unpaired t-test. Scale bar = 50 µm.

Assessing the activation of microglia following peripheral nerve lesion, we observed a vast upregulation of the microglial marker Iba1 in the vicinity of the lesioned motoneurons. The IL/CL ratio for Iba1-IR in C3^−/−^ animals was 5.22±0.27, while the corresponding value for its WT control was 5.06±0.35 (fig. 3A). In K^b−/−^D^b−/−^ mice, the corresponding ratio was 5.39±0.21 compared to its WT control 5.21±0.32 (fig. 3B). No significant differences were detected between the strains (fig. 3C).

**Figure 4:**
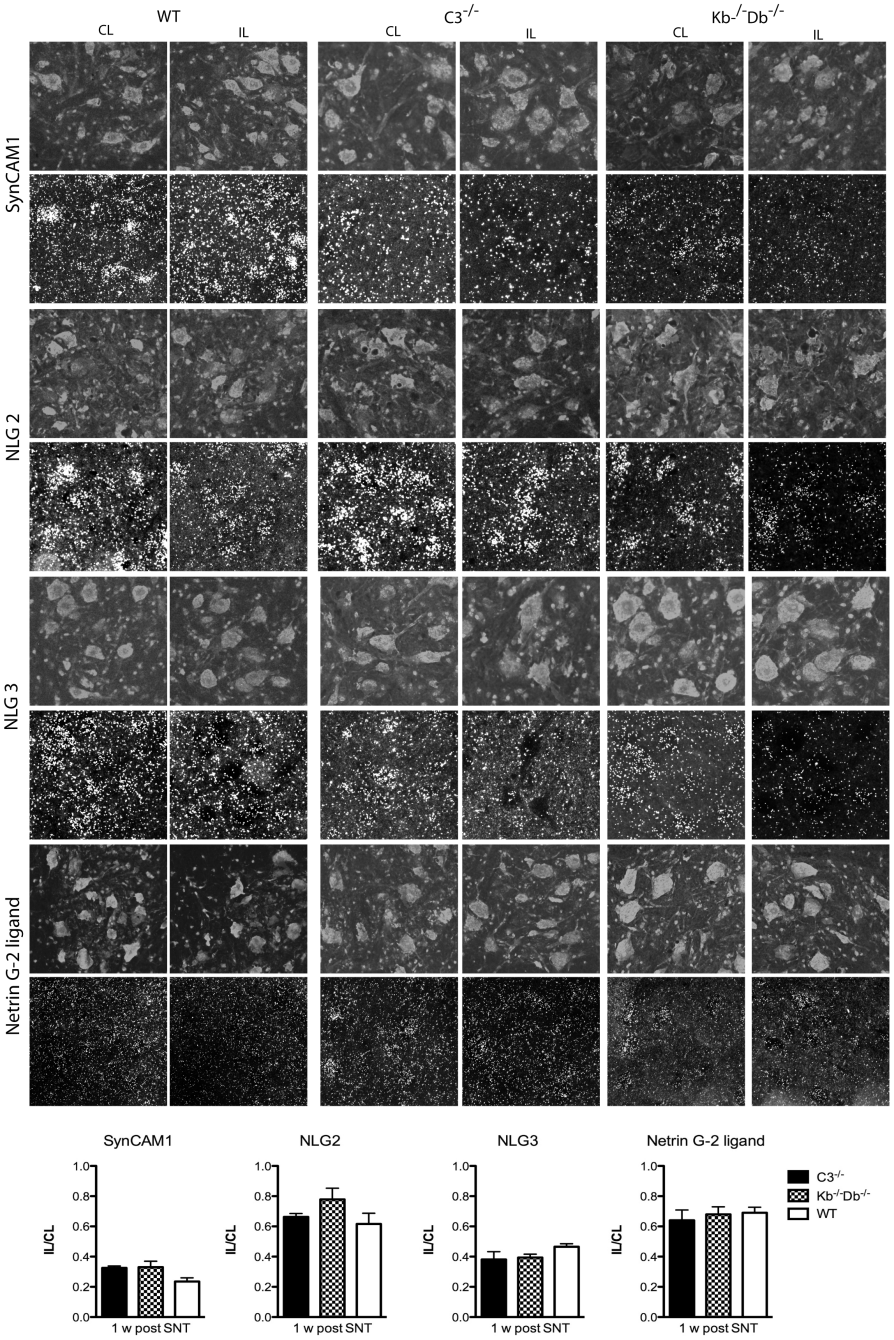
No difference in mRNA signal of SynCAM1, NLG-2, -3 and NGL-2 between WT (first column), C3^−/−^ (second column) and K^b−/−^D^b−/−^ mice (third column) one week after sciatic nerve lesion. ISH signal for SynCAM1 (first row), NLG-2 (second row), NLG-3 (third row) and NGL-2 (fourth row) mRNAs could be seen over motoneuron cell bodies visualized with bisbenzemide in the dorsal part of the ventral horn of the spinal cord. mRNA expression of all studied adhesion molecules decreased after axotomy but no difference was seen between the different mouse lines (quantified in right column ). Error bars indicate SEM, one-way ANOVA with Bonferroni’s multiple comparison test. Five animals were studied in each group. Scale bar = 50 µm.

Finally, we investigated the mRNA regulation of the synaptic adhesion molecules NLG-2, -3, SynCAM1 and NGL-2 after peripheral nerve lesion. The reason for not studying the presence and localization of the proteins with immunohistochemistry rests on the absence of commercially available antibodies for this purpose. For all the studied adhesion molecules we detected a significant decrease in mRNA expression levels in lesioned motoneurons one-week post lesion. Thus, the IL/CL ratio for SynCAM1 was calculated to 0.24±0.06 in WT animals, 0.33±0.09 in K^b−/−^D^b−/−^, and 0.33±0.03 in C3^−/−^ mice. The corresponding values for the NLG-2 IL/CL ratio were 0.62±0.07 in WT, 0.78±0.07 in K^b−/−^D^b−/−^, and 0.66±0.02 in C3−/− mice. For NLG-3, the IL/CL ratios were 0.47±0.02 in WT, 0.39±0.02 in K^b−/−^D^b−/−^, and 0.38± in C3^−/−^ animals. Lastly the IL/CL ratio for NGL-2 mRNA was 0.69±0.04 in WT, 0.68±0.05 in K^b−/−^D^b−/−^, and 0.64±0.07in C3^−/−^ mice (fig. 4). For none of the molecules could a statistically significant difference i.e. IL/CL ratio be detected between the strains.

## Discussion

We here use two mouse strains with increased (K^b−/−^D^b−/−^; [17]) or decreased (C3−/−; [18]) synaptic stripping of axotomized motoneurons to investigate whether the observed differences in the degree of synaptic removal were paralleled by changes in two suggested possible basic events underlying synapse removal [1]; activation of glial cells and/or differences in the regulation of synaptic adhesion molecules in lesioned motoneurons. Surprisingly, these mouse strains, which have very different phenotypes in terms of synaptic stripping, displayed a similar activation of both microglia and astrocytes one week after sciatic nerve lesion, which in turn was very similar to that seen in WT animals. This does not imply that glial cells are not involved in the synaptic elimination, but rather indicates that the gross activation pattern of glia is not correlated to the degree of synaptic stripping. Previous studies using pharmacological blockade of microglial cell proliferation, have argued that microglial cells do not have any influence on the magnitude of synaptic stripping post-axotomy [7].

An alternative explanation for a different synaptic removal in the studied mouse strains may be found on the postsynaptic side, i.e. in the lesioned motoneurons. A major class of molecules with an obvious influence on synaptic formation and maintenance are synaptic adhesion molecules. Thus, we investigated the regulation of NLG-2, -3, SynCAM1 and NGL-2 in motoneurons following peripheral nerve lesion. mRNAs for all these molecules have previously been shown to be downregulated before stripping of synapses takes place, and then return towards control levels as synapses reestablish their connections after successful axonal regeneration of axotomized motoneurons [6], [15], [16]. Also in this case, both K^b−/−^D^b−/−^ and C3^−/−^ mice displayed a response which was very similar to what was seen in WT mice. Thus, a downregulation of synaptic adhesion molecules, at least at the mRNA level, does not by itself infer a downsizing of the synaptic connectivity with lesioned neurons.

Thus, it is evident that the absence of any of the immune molecules classical MHC class Ia and complement protein C3 does not have any effect on glial reactivity, as determined by use of established markers for such reactivity, nor on the downregulation of synaptic adhesion molecules in motoneurons after axotomy. Even if both of these molecules appear to have their main effects on the removal of inhibitory inputs with little effects on excitatory synapses [17], [18], it seems unlikely that this preferential removal would exclude the engagement of surrounding glia. The overall reduction of synaptic removal in C3 deficient animals is massive, since the majority of synapses on the motoneuron are inhibitory [29], [30]. In spite of this dramatic effect on synaptic removal, the glial response is the same. Moreover, the downregulation of synaptic adhesion molecules includes molecules related to both inhibitory and excitatory synapses to the same extent, which makes it very improbable that excitatory synapses are spared by a selective mechanism. Then, how to explain that the absence of the two immune molecules exert strong effects on the removal of synapses from axotomized motoneurons? One obvious possibility is that the effects are mediated through more precise molecular interactions than what can be detected by crude estimates of glial activity or postsynaptic regulation of synaptic proteins. Interestingly, both classical MHC class Ia and C3 are molecules with established functions in the immune system, and it may be asked whether the effects on synaptic connectivity is a result of a general immunological reaction triggered by axotomy. However, also in this context a high degree of precision is present, in that complement C1q protein, which acts upstream C3 in the classical complement cascade, does not seem to exert any effects on the synaptic stripping process [18]. It may then be that MHC class Ia and C3 should be regarded as molecules used by the nervous system to specifically have an influence on synaptic connectivity and plasticity, which is not related to their function in the immune system. This has been proposed both for classical MHC class Ia [31], [32] and for complement proteins [33], [34]. In this context it is of interest that classical MHC class Ia has been shown to be produced by both motoneurons and surrounding microglia in our lesion model [19], while C3 to some extent can be seen in neighboring astrocytes [18].

Thus, classical MHC class I and complement protein C3 are key players exerting an influence on the synaptic removal from motoneurons after lesion. We have shown here that the large differences in the extent of the removal exerted by these molecules are not coupled with differences in the general glial reactive response and/or the downregulation of postsynaptic synaptic adhesion molecules, which have earlier been linked with the synaptic stripping process. Instead, the results indicate the presence of intricate and precise molecular interactions with synaptic inputs destined to be removed after motoneuron injury. Such molecular interactions seem to be at least partly mediated by immune molecules, but the exact mechanisms for the synaptic removal remain elusive.
